# Hidden Industrial Trans-Fatty Acids: Mechanistic Insights into Dyslipidemia, Cardiovascular Disease, and Metabolic Dysfunction-Associated Steatotic Liver Disease

**DOI:** 10.3390/ijms262311715

**Published:** 2025-12-03

**Authors:** Mona A Hegazy, Bojana Vidovic, Shimaa Abobakr, Aleksandra Zeljkovic, Aleksandra Stefanovic, Jelena Vekic

**Affiliations:** 1 Department of Internal Medicine, Faculty of Medicine, Cairo University, Cairo 12556, Egypt; monahegazy@cu.edu.eg; 2 Department of Bromatology, University of Belgrade-Faculty of Pharmacy, 11000 Belgrade, Serbia; bojana.vidovic@pharmacy.bg.ac.rs; 3 Department of Medical Biochemistry and Molecular Biology, Faculty of Medicine, Cairo University, Cairo 12556, Egypt; shimaa.m.abobakr@kasralainy.edu.eg; 4 Department of Medical Biochemistry, University of Belgrade-Faculty of Pharmacy, 11000 Belgrade, Serbia; aleksandra.zeljkovic@pharmacy.bg.ac.rs (A.Z.); aleksandra.stefanovic@pharmacy.bg.ac.rs (A.S.)

**Keywords:** trans-fatty acids, dyslipidemia, cardiovascular disease, MASLD, epigenetics, food policy

## Abstract

Trans-fatty acids (TFAs), particularly industrially produced TFAs (iTFAs), are linked to dyslipidemia, cardiovascular disease (CVD), and metabolic dysfunction-associated steatotic liver disease (MASLD). Despite regulatory efforts, “hidden” TFAs persist in processed foods, posing ongoing health risks. This narrative review synthesizes evidence on the biochemical and metabolic impacts of the most studied TFAs, focusing on dyslipidemia, CVD, and MASLD, and highlights gaps in research and policy. Available data suggest that iTFAs, which are dominant in modern diets, were associated with elevated low-density lipoprotein cholesterol (LDL-C), triglycerides, and lipoprotein (a), reduced high-density lipoprotein cholesterol (HDL-C), exacerbating atherosclerosis, increasing hepatic lipogenesis, oxidative stress, and inflammation and driving MASLD progression to fibrosis, whereas ruminant TFAs (rTFAs) showed neutral or beneficial effects. Epigenetic modifications (e.g., DNA methylation, miRNA alterations) induced by TFAs may further worsen metabolic dysfunction. Analytical challenges and inconsistent food labeling make it difficult to assess TFAs intake. Global disparities in TFAs regulations persist, but some regions still exceed recommended limits. Hidden iTFAs represent a critical public health issue, necessitating stricter policies, improved labeling, and consumer education. Future research should prioritize human studies on TFA-induced epigenetic changes and develop healthier fat alternatives. Eliminating residual iTFAs from the food supply is essential to mitigate cardiometabolic risks globally.

## 1. Introduction

Trans-fatty acids (TFAs) are a form of unsaturated fat where the hydrogen atoms around a carbon–carbon double bond are on opposite sides, known as a ‘trans’ configuration. This can occur in both monounsaturated (MUFA) and polyunsaturated (PUFA) fats. The TFAs can be either of industrial hydrogenation origin, i.e., industrial trans-fatty acids (iTFAs), or naturally from biohydrogenation in ruminant animals known as ruminant TFAs (rTFAs) [1].

The pervasive presence of TFAs in modern diets poses a significant public health concern, despite growing awareness of their detrimental effects. Observational and experimental epidemiological studies have provided robust evidence on the effect of TFAs on human health, in general, and the cardiovascular system. Consumption of iTFAs raises the total high-density lipoprotein cholesterol (HDL-C) ratio in the blood and the risk of coronary heart disease [2,3,4,5].

The effects of rTFAs on lipoproteins and heart diseases are unclear and data on their impact on plasma lipoproteins in humans are limited. Some epidemiological studies have shown no association between rTFAs intake and heart disease risk [2,6]. In contrast, others have shown a non-significant inverse association [7], or a non-significant positive association [3].

While overt sources of TFAs have been increasingly regulated, “hidden” trans-fats, often stemming from partially hydrogenated oils, continue to infiltrate processed foods, creating a subtle yet substantial dietary burden. This insidious intake carries profound implications for metabolic health, particularly concerning serum lipid profiles and the development of cardiovascular disease (CVD) and metabolic dysfunction-associated steatotic liver disease (MASLD), previously called non-alcoholic fatty liver disease (NAFLD).

Understanding the subtle yet significant role of hidden trans-fats is crucial for developing effective dietary interventions and public health strategies. By shedding light on the implications of these often-overlooked dietary components, this study seeks to empower individuals and healthcare professionals to make informed choices that promote metabolic well-being and mitigate the risks associated with chronic disease.

This narrative review discusses the link between hidden trans-fat consumption, dyslipidemia and its associated CVD risk, and MASLD by examining the impact of these concealed TFAs on serum lipid parameters, including low-density lipoprotein cholesterol (LDL-C), HDL-C, triglycerides (TG), and beyond. We aim to elucidate the mechanisms by which TFAs contribute to changes in the serum lipid profile and cardiometabolic risk. Furthermore, we highlight the association between hidden trans-fat intake and the pathogenesis of MASLD, a growing epidemic linked to dietary factors and metabolic dysfunction. Given the limited explicit mention of “hidden” trans-fats in the literature, this review interprets findings on industrially produced TFAs (often found in processed foods) as representative of hidden trans-fats. This approach allows for a discussion of the potential impact of TFAs that are not explicitly labeled or recognized in the diet.

## 2. Summary of the Most Widely Studied Trans-Fatty Acids

The presence of one or more unconjugated trans-double bonds significantly affects the physical, chemical, and biological properties of TFAs compared to the most common cis-unsaturated fatty acids. A more rigid structure and higher melting points make TFAs similar to saturated fatty acids (SFAs) [8]. In vitro and animal studies indicate that TFAs, although less potent than SFAs, promote CVD and other adverse health outcomes [9].

The major iTFAs are elaidic acid, which is a geometrical isomer of oleic acid (C18:1n-9), and linoelaidic acid, as the geometrical isomer of the essential n-6 linoleic acid (C18:2n-6) (Table 1) [10]. Most iTFAs are formed through the partial hydrogenation of vegetable oils, which may contain up to 60% TFAs [11]. Due to their solid consistency and enhanced oxidative stability, hardened vegetable oils, such as margarine and vegetable shortenings, are widely added to improve the texture and shelf life of various food products. Additionally, TFAs can be formed during the refining process of crude oils. It was estimated that processed food and oils contribute about 80% of TFAs in the modern diet [12]. Among these products, commercially baked foods (cakes, cookies, and pies), snacks, and fast foods are commonly considered the primary sources of iTFAs [13]. In addition to industrial processing, thermal food processing at home, such as baking and deep-frying, also contributes to the formation of TFAs. Not only temperature, but other factors such as food constituents (proteins, carbohydrates, and antioxidants), and the material of the frying vessel, affect the formation of TFAs [14,15]. Furthermore, research indicates that cooking with ingredients rich in isothiocyanates and polysulfides, such as garlic, onion, and broccoli sprouts, can increase the formation of the TFAs in edible oils [16].

Certain foods, such as meat, milk, and dairy products, contain natural TFAs produced through the biohydrogenation of PUFAs from feeds in the rumen of animals. However, since ruminant lipids only reach up to 4–6% TFAs, the contribution of ruminant-derived foods to overall TFA intake is minimal compared to highly processed foods [17]. Among natural TFAs, vaccenic acid is the most predominant geometrical isomer, constituting 50–80% of the total rTFAs. It is a precursor of rumenic acid, as the main isomer of conjugated linoleic acid (CLA). This conjugated TFA can also be endogenously formed after ingestion of vaccenic acid from ruminant-derived food by the activity of Δ^9^-desaturase [18]. Among rTFAs, palmitelaidic acid, also known as trans-palmitoleic acid, is proposed as a biomarker of high-fat dairy fat intake. It has also been suggested that it may be endogenously produced from vaccenic acid [19].

At this point, it is important to note that the two main types of fatty acids isomers are geometrical isomers, which differ in their cis or trans configurations, and positional isomers, which differ in the location of the double bond along the fatty acid chain. The formation of iTFAs through industrial partial hydrogenation, deodorization processes, and even certain home-cooking practices can promote the generation of both geometrical and positional trans isomers [14,20]. On the other hand, this specific mixture of positional and geometrical isomers does not occur in some rTFAs. This distinction in isomeric compositions is essential to the divergent biological effects attributed to iTFA and rTFA.

In the human body, TFAs can be formed by geometrical isomerisation due to free radical attack [21,22]. Additionally, there is evidence that certain gut microbes can hydrogenate dietary PUFAs like linoleic acid into various TFAs isomers, including trans-10, cis-12 CLA, which has distinct metabolic effects [23,24]. Therefore, the total “TFA burden” reflects the sum of dietary iTFAs and rTFAs intake together with endogenous TFAs formation, after accounting for TFAs catabolism.

## 3. Impact of Trans-Fatty Acids on Dyslipidemia and Cardiovascular Risk

Dyslipidemia, comprising elevated serum concentrations of total cholesterol (TC), TG, and LDL-C, with decreased levels of HDL-C, is among the major cardiovascular risk factors [25,26,27], and epidemiologic studies have provided convincing evidence of the adverse effects of TFAs on the serum lipid profile. It has been shown that consumption of TFA-rich food is associated with an increase in serum LDL-C, TG, and lipoprotein (a), while also leading to a decrease in HDL-C levels in adults [28,29,30,31]. Similarly, lower total cholesterol (TC) and LDL-C concentrations were observed in parallel with lower consumption of TFAs in children [32]. However, epidemiologic data are not fully complemented by mechanistic explanations of how TFAs modulate lipoprotein metabolism.

### 3.1. Modulation of Lipoprotein Structure and Lipid Metabolism Pathways by Trans-Fatty Acids

**Figure 1:**
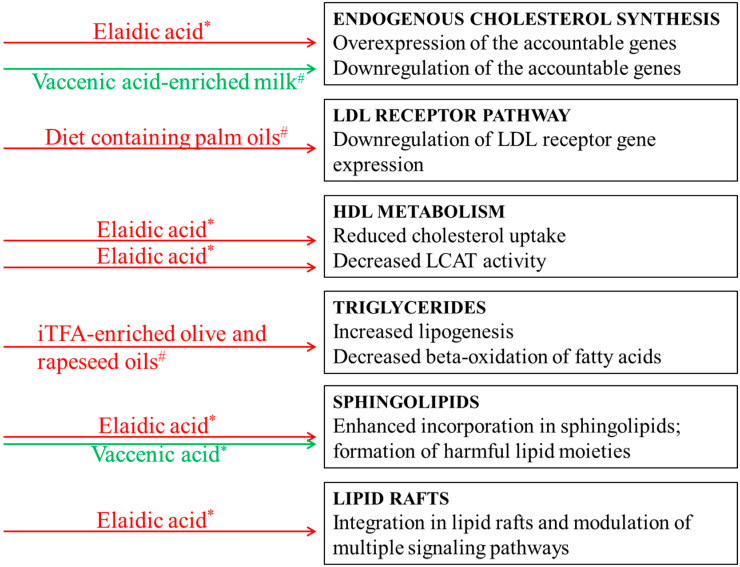
Putative mechanisms of TFAs’ effects on lipid metabolism (* data from in vitro studies; # data from animal studies).

Recent data provide novel insights into the mechanisms by which TFAs influence body lipids (Figure 1). It has been reported that cholesterol synthesis increases when Hepa1-6 cells are treated with elaidic acid. Such an effect is presumably achieved through the overexpression of genes involved in cholesterogenesis [33]. Moreover, iTFAs may contribute to changes in the expression of other genes affecting lipid metabolism. Recent findings demonstrated higher serum elaidic acid levels, in parallel with decreased gene expressions of peroxisome proliferator-activated receptors alpha and gamma and the expression of the LDL receptor, in laying hens fed with different palm oils, compared to hens fed with soybean oil [34].

Yet, it should be emphasized that rTFAs might have divergent effects. It has been reported that vaccenic acid, the most prominent rTFA, does not induce cholesterol synthesis in hepatocytes [35]. A more recent study [36] has shown decreased expressions of genes involved in cholesterol synthesis in the liver of Golden Syrian hamsters fed with milk rich in vaccenic acid. Similarly, a diet rich in vaccenic acid reportedly exhibits favorable effects on the serum lipid profile, insulin sensitivity, and gut microbiome in pigs [37].

In addition to the effects on cholesterol synthesis, TFAs influence other aspects of lipid metabolism. Data obtained from studies, interpreted in accordance with the evolved Bradford Hill considerations, suggest that iTFAs may contribute to a reduction in the expression and activity of LDL receptors [38]. Also, it has been demonstrated that substituting PUFA with iTFAs in olive and rapeseed oils leads to an increase in the liver TG content in mice that are fed these oils. The mentioned effects presumably arise due to the overexpression of lipogenic enzymes and lower expression of mediators of beta-oxidation [39].

Novel evidence suggests that TFAs can alter the metabolic fate of phospholipids and sphingolipids. Recent research shows that iTFAs preferentially incorporate in sphingolipids, promoting their secretion and accelerating very low-density lipoprotein (VLDL) release in mice [40]. Importantly, experiments on cell lines indicated enhanced incorporation of both iTFAs (elaidate) and rTFAs (vaccinate) in ceramides and diacylglycerols, and thereby a formation of harmful lipid moieties with possible deleterious effects [41,42]. It has been suggested that TFAs may act through interactions with lipid rafts [43,44], but further research is required to fully clarify their effects.

### 3.2. Overview of the Impact of Trans-Fatty Acids on Vascular Oxidative Stress and Inflammation

Even though dyslipidemia-related chronic diseases are based on various pathophysiological mechanisms, multifactorial risk assessment pointed out a synergistic interplay of inflammation and oxidative stress as crucial components [45]. Current evidence indicates that excessive diet 5% elaidic acids intake accelerates atherosclerosis by promoting inflammation and oxidative stress in a mouse model of hyperlipidemia [46]. Comprehensive research of the mechanism of the inflammatory effect of TFAs has been performed, in vitro, in different cell models. It has been shown that TFAs integrate into the membranes of adipocyte, monocyte, and macrophage cells, amplifying inflammatory signaling pathways [47]. Evidenced by previous research, elaidic and linoelaidic acids activate endothelial NF-κB signaling and reduce NO in human microvesicular endothelial cells [48]. Furthermore, iTFAs promoted DNA damage-induced apoptosis by inducing mitochondrial oxidative stress [49] and inhibited cell growth by activating oxidative stress in the endoplasmic reticulum and unfolded protein response signaling pathways in SH-SY5Y cells [50]. Importantly, available data indicate that the inflammatory effects are related to iTFAs, while rTFAs have no impact or may even exhibit anti-inflammatory properties [9]. In line with this, research by Lee et al. [51] showed on isolated mice splenocytes that dietary trans-11 18:1 vaccenic acid at physiological concentrations of 50–200 μM enhanced the IL-10 and suppressed TNF-alpha production.

### 3.3. Human Studies Assessing the Link Between TFAs, Dyslipidemia, Oxidative Stress, Inflammation, and Cardiovascular Risk

Ongoing research continues to assess the broader health implications of TFAs consumption (Table 2), as regulatory compliance remains incomplete [52], particularly in Southeast European countries [53] and in the Eastern Mediterranean Region [54]. There is an ongoing debate about whether rTFAs and iTFAs have similar cardiometabolic effects.

Although a body of evidence confirms the contribution of TFAs to the altered serum lipid profile, oxidative stress, and inflammation, it should be noted that much of the available data comes from in vitro and animal studies exploring the effects of single, purified TFAs on specific bioactive lipid compounds. However, considering the complexity of the human diet and the diversity of both ingested [14,20] and endogenously formed TFAs [18,24], these findings can only serve as indicators of the potential effects. A complete understanding of putative effects of TFAs in humans requires further studies designed to fully appreciate the multi-faceted nature of human exposure to these compounds.

In line with the findings of cell culture and animal studies, a recent cohort study conducted in the elderly Norwegian population recorded inverse associations between rTFAs serum levels and LDL-C and TG concentrations, while direct associations were found with HDL-C [55]. Yet, it should be noted that findings from human studies are not fully consistent. For instance, significantly higher plasma TC and LDL-C levels were recorded in healthy volunteers who adhered to a 4-week diet with alpine butter as a source of rTFA [56]. Noteworthy, such an elevation of serum cholesterol concentrations might arise due to the joint influence of other lipid components of butter, instead of the effect of rTFA solely. Similarly, a systematic review by Verneque et al. [57] indicated that both rTFA and iTFA intakes are associated with worsened TC and LDL-C levels, but have inverse effects on HDL-C levels. Yet, the authors emphasized that the total effect of TFA intake depends on the dose, but also on the composition of ingested food.

Iino et al. [58] have reported that the increased proportion of elaidic acid in the phospholipids of HDL negatively affects the cholesterol uptake capacity in human sera by decreasing the fluidity of the HDL surface and the activity of lecithin-cholesterol acyltransferase (LCAT). Interestingly, it was recently shown that whole TFA intake can have variable effects on insulin sensitivity in adult carriers of distinct phenotypes of the cholesteryl ester transfer protein gene (*CETP*), suggesting a possible influence of TFAs on CETP activity [59]. In line with this, it was reported that overall levels of mono TFAs (trans-palmitoleic, elaidic, and vaccenic) positively correlate with TG levels in human serum [60]. Epidemiological studies reported that the distinct TFA content of plasma phospholipids may be related to changes in body mass index, although findings remain inconsistent [61,62].

Human randomized trials that replace other fats with iTFA-rich partially hydrogenated oils confirm a systemic pro-inflammatory effect, validating that the effects of pure compounds translate when consumed as part of a complex food [63]. Two separate cross-sectional studies showed that iTFAs were associated with significantly high plasma inflammatory markers, such as TNFα, IL-6, C-reactive protein (CRP), and chemokine (C-C motif) ligand 2 (CCL2) in overweight women [64] and in cardiac patients [65]. In healthy men, a diet containing 8% of daily energy from iTFA increased CRP levels, whereas a soybean oil-based stick margarine-rich diet raised TNFα, IL-1β, and IL-6 production in the peripheral blood mononuclear cells of hypercholesterolemic subjects [9]. In CVD patients, TFAs levels correlated with parameters of oxidative stress, especially final lipid oxidation products [66]. Furthermore, a recent epidemiologic study has demonstrated a negative association between dietary trans-C18:1 (n-7) intake and adiposity, diabetes, and inflammation [67]. Despite this, due to limited long-term epidemiologic studies and the potential lipid-modifying effects of rTFAs at high doses, further controlled studies are needed to confirm their anti-inflammatory effects.

Chandra et al. [55] reported that both ruminant and industrial TFAs in plasma were associated with a favorable CVD risk profile; another study showed that in type 1 diabetes patients, higher all-C18:1trans isomers in erythrocyte membranes correlated with increased carotid plaques [68]. Notably, all-C18:1trans are prevalent TFAs found in partially hydrogenated vegetable oils and ruminant fats. Lechner et al. [69] found that naturally occurring trans-palmitoleic acid levels in heart failure patients were linked to a better cardiometabolic risk profile, whereas iTFAs were associated with dyslipidemia. In contrast, NHANES data showed that plasma TFAs, regardless of origin, were positively associated with elevated NT-proBNP, a biomarker of heart failure [70].

In type 2 diabetes, higher dairy intake increased trans-palmitoleic acid levels and correlated with TC and TG levels, but not with changes in body weight or metabolic control [71]. Yet, a meta-analysis on dairy fat intake found no association between circulating trans-palmitoleic acid and CVD incidence or mortality [72]. The observed differences between the findings may arise from dietary variations in the amount, confounding variables, or lack of harmonization between analytical methods for fatty acid determination in biological samples. Although comparative studies are limited, rTFAs are generally considered less harmful due to their lower dietary intake. Nevertheless, additional data are needed to clarify their respective roles in CVD and other metabolic disorders.

**Table 2:** Overview of recent studies assessing the link between TFAs and cardiovascular risk.

Study	Study Design	Study Population	Assessment of TFAs Intake	Assessed CVD Risk Factors or Outcomes	Main Findings
Riley et al., 2024 [31]	Meta-analysis	Subgroup analysis of 8 randomized controlled trials including 334 healthy or at-risk participants	Intake of partially hydrogenated vegetable oils	Levels of Lp(a)	Replacement of saturated fatty acids with TFAs led to a significant increase in Lp(a) levels.
Chandra et al., 2020 [55]	Cross-sectional study	3706 subjects from Norway	Intake assessed by plasma fatty acid composition	Lipid profile, body mass index, systolic and diastolic blood pressure	Both ruminant and industrial TFAs levels in plasma were associated with favorable CVD risk profile.
Mesa et al., 2021 [68]	Cross-sectional study	167 patients with type 1 diabetes mellitus from Spain	Intake assessed by fatty acids composition of erythrocyte membranes	Presence of carotid plaques	Positive association between all-C18:1trans levels in the erythrocyte membranes and the presence of ≥3 carotid plaques.
Lechner et al., 2023 [69]	Prospective cohort study	404 patients with heart failure with preserved ejection fraction in Aldo-DHF trial from Germany and Austria	Intake assessed by whole blood fatty acid composition	Cardiometabolic risk factors, aerobic capacity, and cardiac function	Higher levels of industrial TFAs were associated with increased LDL-C, TG, and HbA_1c_ levels and lower aerobic capacity. Natural TFAs were inversely associated with cardiometabolic risk factors.
Wang et al., 2025 [70]	Cross-sectional study	1478 participants of the NHANES study from US	Intake assessed by plasma fatty acid composition	Levels of NT-proBNP	Higher levels of major TFAs in plasma (elaidic, vaccenic, and linolelaidic acids) and their sum were positively associated with elevated NT-proBNP.
Mitri et al., 2021 [71]	Randomized clinical trial	111 participants with type 2 diabetes mellitus from US	Intake from dairy products	Metabolic control, body weight, and CVD risk factors	Increased dairy consumption was linked to higher trans-palmitoleic acid levels, which positively correlated with TC and TG levels.
Trieu et al., 2021 [72]	Meta-analysis	Subgroup analysis of 6 studies including 3477 participants	Intake from dairy products	CVD incidence and all-cause mortality	No association between trans-palmitoleic acid levels and CVD risk and all-cause mortality.
Zhu et al., 2019 [73]	Meta-analysis	744,736 subjects from 24 studies	Intake assessed by food frequency questionnaire, 7-day weighed food record or 24 h dietary recall	CVD incidence or mortality	A positive dose–response association was found between dietary TFAs intake and CVD risk.
Ivey et al., 2023 [74]	Prospective cohort study	158,198 participants in the Million Veteran Program from US	Intake assessed by food frequency questionnaire and estimated total fatty acid composition of commonly consumed oils and fats	Atherosclerotic CVD event (ischemic heart disease, ischemic cerebrovascular disease, and peripheral artery disease)	Higher intakes of monounsaturated TFAs and rumenic acid were linked to increased risks of ischemic heart disease and peripheral artery disease.
Zeinalabedini et al., 2024 [75]	Case–control study	443 patients with ischemic heart disease and 453 controls from Iran	Intake assessed by food frequency questionnaire	Ischemic heart disease	Intake of TFAs was not associated with ischemic heart disease.
Yao et al., 2021 [76]	Prospective cohort study	101,832 subjects from PLCO cancer screening trial from US	Intake assessed by food frequency questionnaire	All-cause, CVD, and cancer mortality	Dietary intake of TFAs was associated with all-cause mortality, but not with CVD and cancer mortality.

Abbreviations: TFAs, trans-fatty acids; CVD, cardiovascular disease; Lp(a), lipoprotein (a); LDL-C, low-density lipoprotein cholesterol; TG, triglycerides; TC, total cholesterol; HbA_1c_, glycated hemoglobin; NT-proBNP, N-terminal pro B-type natriuretic peptide.

A meta-analysis by Zhu et al. [73] found a dose-dependent link between TFAs intake and CVD risk. A prospective study of the US veterans demonstrated that participants in the highest quintile of total monounsaturated TFAs intake had 13% higher CVD risk than those in the lowest quintile [74]. In contrast, a recent Iranian case–control study found no association between TFAs intake and ischemic heart disease [75], suggesting that substantial regional differences in TFAs consumption might affect their link with CVD. Increased TFAs consumption is thought to increase the risk of CVD mortality [77]. Yao et al. [76] found a modest association between TFAs intake and all-cause mortality, but no link with CVD or cancer mortality. Of note, these studies relied on food frequency questionnaires to estimate TFAs intake, which do not specify individual fatty acids and therefore could be affected by misclassification or inaccurate assessment. Analysis of randomized controlled trials and prospective studies showed a modest increase in mortality risk with TFAs intake [78]. Strong evidence supporting this hypothesis is provided by data from Denmark, where efforts to reduce iTFAs in food contributed to an 11% reduction in CVD mortality between 1991 and 2007 [79].

When evaluating the effects of TFAs under specific health conditions, such as CVD, obesity, or other metabolic disorders, it is important to distinguish between iTFA, rTFA, and those generated endogenously. These conditions involve distinct levels of free radical production and oxidative stress contributions to metabolism. This complexity makes it exceptionally difficult to discern the role of TFAs derived from “hidden industrial sources” from those derived from endogenous isomerization of natural cis fatty acids, caused by free radical species. Indeed, in humans, the literature indicates that oxidative stress can promote the formation of trans isomers from both MUFA and PUFA in cardiovascular and other diseases [21]. Consequently, measured TFA levels in human specimens (plasma, tissues) cannot be univocally attributed to dietary intakes alone, as discussed above. The contribution of endogenous TFAs, which are not obtained from the diet, can be identified by specific biomarkers, such as palmitelaidic acid [80].

## 4. Impact of Trans-Fatty Acids on Hepatic Steatosis, Fibrosis, and MASLD Progression

MASLD encompasses a wide variety of histopathological images, ranging from simple steatosis to inflammation, hepatic injury, fibrosis, end-stage hepatic disease, and hepatocellular carcinoma [81]. The multiple-hit theory describes the multifactorial pathogenesis of MASLD [82]. Dietary habits, genetic and epigenetic factors, obesity, insulin resistance, disturbed intestinal microbiota, adipocyte proliferation, adipocyte dysfunction, inflammation, oxidative stress, and mitochondrial dysfunction are all thought to participate cooperatively in the development and progression of MASLD [83].

Numerous animal studies have been conducted to elucidate the mechanisms by which TFAs are linked to MASLD, but previously published human studies are still limited. Therefore, it is crucial to highlight the magnitude of the outcome behind using TFAs, especially those industrially produced. Here, we highlight the possible principal links between TFAs and MASLD.

### 4.1. Modulation of Hepatic Lipid Metabolism by Trans-Fatty Acids

Hepatocyte TG content is affected by the balance between fatty acids input, which involves their uptake, synthesis, and esterification by liver cells, and output, which includes fatty acids oxidation and TG export [84]. In a comparative study on mice, TFAs intake was associated with increased hepatic TG, a key hallmark of MASLD, through the augmented stimulation of hepatic de novo lipogenesis and inhibition of hepatic lipolysis [85].

Mechanistic insights into the accumulation of TG in liver cells and TFA-induced hepatic steatosis often originate from reductionist models. For instance, treatment with pure elaidic acid upregulates key lipogenic enzymes, such as acetyl-CoA carboxylase, fatty acid synthase, stearoyl-CoA desaturase-1, and 3-hydroxy-2-methylglutaryl-CoA reductase, in hepatocytes and animal models, providing direct evidence of this pathway [86]. The physiological relevance of this mechanism is strengthened by studies using complex diets high in partially hydrogenated oils, which also result in hepatic TG accumulation, demonstrating that the lipogenic effect of industrial TFAs is robust enough to manifest within a complex food matrix [82]. Additionally, the inhibition of adipose triglyceride lipase (ATGL), the primary enzyme responsible for breaking down TG in the liver, further contributes to TG buildup [82]. Together, these mechanisms promote excessive fat storage within liver cells.

Additionally, insulin signaling pathways like insulin receptor substrate 1, Protein kinase C, and Ak strain transforming were suppressed in murine liver cells by TFAs, indicating hepatic insulin signaling impairment, which reflects insulin resistance and aggravates hepatic steatosis [87].

### 4.2. The Impact of Trans-Fatty Acids on Hepatic Oxidative Stress and Inflammation

Oxidative stress develops when the balance between reactive oxygen species (ROS) production and antioxidant defense is disrupted. Not only are TFAs linked to increased ROS production through impaired mitochondrial function or increased endoplasmic reticulum stress [88], but they also result in downregulated hepatic antioxidant enzymes such as peroxidase, thiobarbituric acid reactive substances, catalase, superoxide dismutase, and glutathione [89].

In addition, TFAs are robustly linked to increased hepatic inflammation [81], as reflected by hepatic NF-κB activation, high TNFα expression, osteopontin, CCl2, and macrophage markers [90]. Moreover, high liver IL-1β levels were linked to high iTFAs intake in mice [91]. Furthermore, in media containing TFAs compared to media with cis isomers, TNFα production was increased from the Kuffer Cells (KCs). So, this pathophysiological result can be explained partially by pro-inflammatory cytokine production and phagocytosis of macrophages and KCs [92].

### 4.3. Trans-Fatty Acids and Hepatic Fibrosis

Previous studies have linked iTFAs intake to the occurrence and progression of MASLD; however, the exact mechanism is unknown. An earlier study revealed that non-alcoholic steatohepatitis was induced by a high TFA diet [93]. This result was augmented by a comparative study in rats that were fed with different fatty diets and high-TFA diets, which induced more severe liver steatosis [94]. Similarly, compared to mice fed with cis and saturated fat diets, a TFA diet was associated with more advanced MASLD, high ALT, high hepatic TG and cholesterol levels, and increased expression of fibrosis-related genes [34], so the evidence for progression to fibrosis comes largely from animal models fed semi-purified diets spiked with specific iTFAs. These studies show that, in isolation, these compounds are sufficient to drive pro-fibrotic gene expression. Another animal study found that when TFAs were added to an atherogenic diet, it resulted in a more steatotic phenotype [94]. Moreover, a diet rich in TFAs endorses liver tumorigenesis, especially in HCV-infected mice. This could occur via the persistent stimulation of different inflammatory pathways [95].

### 4.4. Changes in Hepatic Function Induced by Trans-Fatty Acids

As observed in mice fed with TFAs, TFAs impair liver functions, which are indicators of hepatic injury [34,63,92]. Also, TFAs were linked to decreased microsomal triglyceride transfer protein (MTP) mRNA, leading to inadequate TG transfer to nascent ApoB particles. This led to a diminished VLDL secretion [88].

### 4.5. Human Studies Assessing the Link Between TFAs, MASLD, and Lipoproteins

As summarized in Table 3, a limited number of human studies have investigated the association between TFAs and MASLD.

**Figure 2:**
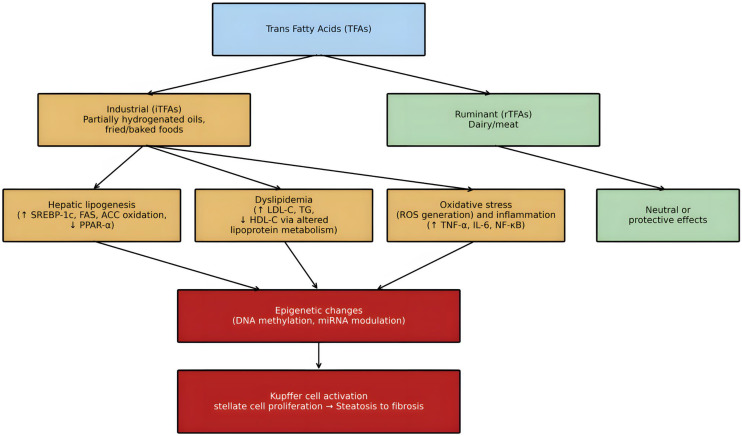
Proposed mechanisms linking TFAs to dyslipidemia and MASLD. Mechanistic pathways through which TFAs contribute to dyslipidemia and MASLD. Industrial TFAs promote hepatic lipid accumulation, oxidative stress, and inflammation, driving MASLD progression. Ruminant TFAs may have neutral or protective effects.

While Lechner et al. [69] have mentioned an opposite association between plasma rTFAs and MASLD markers, Kratz et al. [98] referred to an improvement in glucose tolerance with dairy fat intake, possibly through reducing hepatic fat and improving liver and systemic insulin sensitivity. Regarding iTFAs, according to the Global Burden of Disease Data 2017, a major related risk factor of MASLD liver deaths was a high intake of TFAs [96]. Furthermore, a human study involving 4252 contributors showed a positive stepwise association between TFAs and the possibility of MASLD development. Higher TFA levels were associated with a worsened liver biochemical profile and an increased fatty liver index (FLI), indicating a higher possibility of MASLD and acceleration of its progression [97]. Additionally, a previous study demonstrated that TFAs were associated with high plasma levels of primary bile acids, which correlated with impaired cholesterol homeostasis [100]. MASLD patients had higher plasma eliadic acid levels, showed high fasting insulin, total cholesterol, and lower HDL-C, as well as higher hepatic TG content and a disturbed FA composition of hepatic and abdominal TG. They also exhibited considerably higher oxidative stress-related parameters in the liver [99]. The proposed mechanisms linking TFAs to dyslipidemia and MASLD are illustrated in Figure 2.

As noted above, an interpretation of TFAs’ effects in human studies on MASLD requires a clear distinction between iTFA, rTFA, and those endogenously formed. Given that MASLD patients exhibit enhanced oxidative stress, it becomes difficult to differentiate TFAs originating from industrial sources from those generated in vivo through free radical-driven isomerization of natural cis fatty acids. Consequently, TFAs levels measured in plasma cannot be attributed solely to dietary exposure.

## 5. The Issue of Hidden Trans-Fatty Acids and Food Labeling Regulations

Since the 1960s, the rising popularity of margarine and vegetable shortenings as cost-effective and healthier alternatives to animal lipids has resulted in estimated TFAs accounting for 2–3% of the total energy intake [7]. Due to their atherogenic potential, excessive intake of TFAs has been recognized as a significant and preventable factor in diet-related non-communicable diseases [101]. Therefore, over the past 20 years, different strategies have been applied to remove industrial TFAs from the food supply [102]. In 2010, the mean global intake was 1.4% of total energy intake [103], which exceeded the recommended limit of <1% of total energy intake [104]. According to the most recent epidemiological data, the global TFAs intake was below 0.3 to 4.2% of the total energy intake, indicating substantial reductions and the effectiveness of policies for reducing industrial TFAs [1].

In essence, “hidden TFAs” are industrially produced trans-fats that consumers unknowingly ingest because they are not clearly labeled as “trans-fat” due to regulatory loopholes, they are disguised under technical ingredient names like “partially hydrogenated oil”, or they are prevalent in foods that do not carry nutrition labels, such as restaurant meals, bakery items, and fried street foods. This “hidden” nature poses a critical public health challenge, as individuals trying to make healthy choices may still be consuming significant amounts of harmful iTFAs, contributing to dyslipidemia, cardiovascular disease, and MASLD without their knowledge. Several studies have reported that many foods, such as pastry [105] and cream-filled pastries [106], biscuits [107,108], savory baked goods [109], and fast foods (e.g., pizza, hot-dog, burger, fries, and pancakes) [10] exceeded regulatory TFA limits.

Despite the World Health Organization (WHO) setting the elimination of iTFAs from the global food supply as a priority by the end of 2025 [110], in some regions, challenges in this task still exist [111]. Although margarines were among the first successfully reformulated food products [11], a recent study reported that in Kazakhstan, margarine and spreads had on average more than 10% TFAs and that only 11% of these products, released in the market between 2018 and 2021, had less than 2% TFAs [112]. Also, a recent study has highlighted that two-thirds of branded soybean oils in Bangladesh exceeded the TFA limit [113]. On the contrary, although exposure to TFAs from edible oils in China is low, there is a recommendation that most consumed soybean oil should be replaced with other vegetable oils, such as safflower, sunflower, and rapeseed oil [114]. Among different food categories, fast food, canned and frozen products, and confectionery were identified as the major contributors to the TFAs in Egypt, the country with the highest TFAs intake [111]. Although data on the occurrence and estimated TFAs intake in Serbia are scarce, some previous studies have reported that processed foods, such as salami, wafers, tea biscuits, and snack products, can elevate the burden of trans-fat in diets, especially among young adults [115,116].

Regulatory approaches to reduce industrial-produced TFAs intake are based on mandatory labeling of TFAs, setting TFAs limits in the food products (2 g per 100 g of fat), and banning the use of partially hydrogenated oils. Due to the persistent presence of TFAs, particularly in processed and fried foods, continuous monitoring, mandatory regulatory measures, and public health education are needed to minimize the risk of further elevated iTFAs intake. Given the decline in the intake of iTFAs and the fact that rTFAs now serve as the primary dietary source of TFAs (accounting for 0.1 to 0.7% of energy intake) for over two-thirds of countries, it is important to promote dietary patterns that limit the consumption of full-fat dairy products and high-fat meats [1]. In addition to hidden TFAs that occur naturally in foods, awareness should be raised about the TFAs formed through traditional cooking methods and the monitoring of TFAs in non-prepackaged foods and trans-fat replacements [117].

## 6. Future Directions

Despite global progress in reducing dietary exposure to industrially produced TFAs, some regions need further support to implement national policies to eliminate these undesirable ingredients from the diet. In addition to enforcing stronger regional policies and monitoring TFAs in non-prepackaged and restaurant foods, nutritional education strategies should be used to raise public awareness on the risks of TFAs intake and the benefits of promoting healthy dietary choices. Considering the shift towards a plant-based diet and the importance of trans-fat replacements in the food industry, further research should focus on innovative technologies and the production of sustainable trans-fat substitutes and studying their long-term health effects.

To gain further insight into TFAs’ activities and health effects, robust and reliable methods for their assessment are of particular importance. Gas chromatography (GC) is the preferred method for analyzing TFAs’ profiles after their conversion to methyl esters by acid- or base-catalyzed reactions. Volatile TFA methyl ester can be detected using a flame ionization detector (FID) or a mass spectrometer (MS) [118]. Yet, different procedures for the optimization of chromatographic conditions and variable types of used detectors are the main factors that limit the comparison among the studies. Further, the same molecular mass and chemical structure of cis and trans fatty acids isomers require a high resolution for their precise chromatographic separation. The next analytical challenge in TFAs determination is the wide range of their concentrations. At present, GC–MS methods offer better analytical performance for accurate quantification. The most common method for TFAs determination in human blood was based on GC with non-specific FID, and the results are presented as a percentage of each detected fatty acid. Recently, a new isotope dilution–gas chromatography–negative chemical ionization–mass spectrometry method for the quantitation of four major TFAs in human blood was validated with an adequate sensitivity and specificity [119]. High-performance liquid chromatography techniques (HPLC) and capillary electrophoresis coupled with MS can also be used for TFAs profiling, but these methods have not reached significant usage in laboratory practice [120]. Future research should focus on the standardization of analytical protocols across laboratories, the improvement of the resolution for isomer separation, and the implementation of highly sensitive and specific MS-based approaches. Such methodological improvements are essential to ensure reliable inter-study comparability and to clarify the role of TFAs in lipoprotein metabolism, and consequently, their contribution to CVD risk and MASLD.

Future studies on the effects of TFAs on human health should go in two directions. One is to definitively resolve the old dilemmas regarding the specific role of industrial and ruminant TFAs. The other is to gain detailed insights into the effects of TFAs on specific lipid molecules and molecular pathways. Comprehensive answers to these questions might form the basis for the potential use of the serum levels of certain TFAs as non-invasive biomarkers of specific metabolic changes, or as a precision medicine tool for the prevention and risk management of cardiometabolic diseases. In line with this, emerging research suggests that TFAs may induce epigenetic modifications such as DNA methylation, which regulate gene expression in response to environmental factors, particularly diet. It has been demonstrated that elaidic acid affects DNA methylation in THP-1 cells, leading to the upregulation of pro-inflammatory genes and the suppression of anti-inflammatory genes [121]. In C57BL/6 mice, maternal elaidic acid intake increased DNA methylation in offspring adipose tissue [121]. These findings suggest that maternal TFAs consumption may induce epigenetic changes in offspring, potentially increasing the risk of metabolic disorders later in life [122]. TFAs may also modulate microRNA expression (miRNA) [123], and a TFA-rich diet alters HDL-associated miRNAs linked to lipid metabolism in healthy men [124]. However, most studies on TFA-induced epigenetic changes have been conducted in cell cultures or animal models, with limited clinical data available. Hence, future research is required to clarify the clinical significance of the observed epigenetic effects.

This review highlights the adverse effects of hidden TFAs on dyslipidemia, cardiovascular risk, and MASLD, emphasizing their impact on lipoprotein metabolism and hepatic lipogenesis. However, several limitations should be considered. Most evidence on TFAs and MASLD is derived from animal or cell studies, with few direct human clinical trials, which limits the generalizability of the findings to human populations. Many studies focus on labeled TFAs, but unlabeled or naturally occurring TFAs (e.g., from frying oils) are often overlooked, leading to a potential underestimation of actual intake. While regulatory progress has been made, inconsistent labeling results in TFAs remaining hidden and undeclared in many foods. Additionally, TFAs are frequently consumed alongside other unhealthy dietary components (e.g., sugars, refined carbohydrates), making it challenging to isolate their independent effects on dyslipidemia and MASLD. Finally, regional disparities in TFAs consumption further limit the universal applicability of the findings, as dietary habits, food regulations, and TFAs sources vary across countries.

## 7. Conclusions

TFAs in processed foods represent a significant public health concern due to their detrimental effects on lipid metabolism and their role in MASLD. Despite regulatory efforts to reduce iTFAs, their persistence in foods such as baked goods, fast food, and fried products continues to contribute to dyslipidemia, characterized by elevated LDL-C, triglycerides, and reduced HDL-C. Additionally, TFAs promote hepatic lipogenesis, oxidative stress, and inflammation, thereby accelerating the progression of MASLD to fibrosis and liver dysfunction. While rTFAs may have neutral or even beneficial metabolic effects, iTFAs are consistently linked to adverse health outcomes. Emerging evidence suggests that TFAs induce epigenetic modifications, influencing gene expression related to inflammation and lipid metabolism. However, most findings are derived from animal and cell studies, highlighting the need for further clinical research.

## 8. Recommendations

To mitigate risks, stronger global policies, improved food labeling, and public awareness campaigns are essential. Future research should focus on developing healthier fat alternatives and clarifying the long-term effects of TFAs substitutes. Addressing hidden TFAs in the diet is crucial for preventing metabolic disorders and improving liver and cardiovascular health worldwide by the following key strategies:

- Strengthen regulations to eliminate iTFAs from processed and restaurant foods.
- Enhance food labeling to improve consumer awareness of hidden TFAs.
- Promote dietary education on the risks of TFAs and healthier fat alternatives.
- Advance research on TFA-induced epigenetic changes and precision nutrition strategies.

By implementing these measures, the global burden of TFAs on metabolic health can be significantly reduced.

## Figures and Tables

**Table 1:** Structures and origin of the most studied TFAs.

Name	Structure	Origin
Elaidic acid	C18:1n-9t	Industrial TFA
Linoelaidic acid	C18:2n-6tt	Industrial TFA
Palmitelaidic acid	C16:1n-7t	Ruminant TFA
Vaccenic acid	C18:1n-7t	Ruminant TFA
Rumenic acid	C18:2n-9c, 11t	Ruminant TFA

**Table 3:** Overview of studies assessing the link between TFAs, MASLD, and lipoproteins.

Study	Study Design	Study Population	Source of TFAs	Assessment of MASLD	Main Findings
Lechner et al., 2023 [69]	Cohort study	404 heart failure patients with preserved ejection fraction (HFpEF) have a mean age of 67 years, with 52% being female	Naturally occurring and iTFAs	Liver enzymes (ALT and GGT)	Plasma naturally occurring TFAs were inversely associated with dyslipidemia, BMI, MASLD markers, and inflammation. iTFAs were positively associated with dyslipidemia and dysglycemia.
Paik et al., 2022 [96]	Data collected from the 2017 GBD (global population-level retrospective study)	Population-level retrospective risk–outcome analysis (using Global Burden of Disease data)	All sources, including ruminant products and partially hydrogenated vegetable oils	Variable due to the different countries where the data are collected	MASLD liver deaths were 2.3 per 100,000 (2017) and correlated with a high intake of sugar-sweetened beverages, red meat, TFAs, and a low intake of nuts/seeds and milk, IKF, increased BMI, FG, and BP.
Mazidi et al., 2018 [97]	Cross-sectional study	A total of 4252 participants, comprising 46.4% men. The mean age was 50.6 years	iTFAs	Liver tests and FLI	Positive significant associations between TFA levels and the possibility of MASLD determined by FLI, liver tests, and BMI.
Kratz et al., 2014 [98]	Cross-sectional study	17 patients with MASLD and 15 controls matched for age and BMI	Naturally occurring TFAs	By either liver biopsy within only the past 3 years or elevated liver enzymes combined with fatty liver by US or CT after other causes of liver dysfunction were excluded	Dairy fat, including naturally occurring TFAs, was in inverse association with FG and hepatic fat content, while it had a positive association with systemic and liver insulin sensitivity.
Araya et al., 2004 [99]	Cohort study	30 patients with a BMI of 45.6 ± 8.3 kg/m^2^ and with age range of 39–45 years, who had voluntary therapeutic gastroplasty or gastrectomy. The control group consisted of those who underwent voluntary anti-reflux surgery	iTFAs	Haematoxylin/eosin-stained liver sections	MASLD patients exhibited a significantly high elaidic acid level, were significantly more obese, and had higher fasting insulin levels, higher plasma TC, lower HDL-cholesterol levels, higher hepatic TG content, a disturbed FA composition in hepatic and abdominal TG, and a higher lipid peroxidation index.

Abbreviations: TFAs, trans-fatty acids; HFpEF, heart failure patients with preserved ejection fraction; ALT, alanine-aminotransferase; GGT, gamma-glutamyl transferase; BMI, body mass index; MASLD, metabolic dysfunction-associated fatty liver disease; iTFAs, industrial TFAs; GBD, Global Burden of Disease; IKF, impaired kidney function; FG, fasting glucose; BP, blood pressure; FLI, fatty liver index; US, ultrasonography; CT, computed tomography; TC, total cholesterol; TG: triglycerides.

## Data Availability

No new data were created or analyzed in this study. Data sharing is not applicable to this article.

## Abbreviations

The following abbreviations are used in this manuscript:

TFAs	Trans-fatty acids
MUFAs	Monounsaturated fatty acids
PUFAs	Polyunsaturated fatty acids
iTFAs	Industrially produced trans-fatty acids
rTFAs	Ruminant trans-fatty acids
CVD	Cardiovascular disease
MASLD	Metabolic dysfunction-associated fatty liver disease
TC	Total cholesterol
LDL-C	Low-density lipoprotein cholesterol
HDL-C	High-density lipoprotein cholesterol
TG	Triglycerides
CETP	Cholesteryl ester transfer protein
LCAT	Lecithin-cholesterol acyltransferase
CRP	C-reactive protein
ROS	Reactive oxygen species
FLI	Fatty liver index
GC	Gas chromatography
miRNA	Micro RNA
